# Immunization with recombinant enterovirus 71 viral capsid protein 1 fragment stimulated antibody responses in hamsters

**DOI:** 10.1186/1743-422X-9-155

**Published:** 2012-08-09

**Authors:** Wei-Choong Ch’ng, Eric J Stanbridge, Kum-Thong Wong, Kien-Chai Ong, Khatijah Yusoff, Norazizah Shafee

**Affiliations:** 1 Department of Microbiology, Faculty of Biotechnology and Biomolecular Sciences, Universiti Putra Malaysia, 43400, UPM, Serdang, Malaysia; 2 Department of Microbiology and Molecular Genetics, School of Medicine, University of California, Irvine, USA; 3 Department of Pathology, Faculty of Medicine, University of Malaya, Kuala Lumpur, Malaysia; 4 Department of Molecular Medicine, Faculty of Medicine, University of Malaya, Kuala Lumpur, Malaysia; 5 Institute of Biosciences, Universiti Putra Malaysia, 43400, Serdang, Malaysia

**Keywords:** EV71, Recombinant protein, Hamster, Immune response

## Abstract

Enterovirus 71 (EV71) causes severe neurological diseases resulting in high mortality in young children worldwide. Development of an effective vaccine against EV71 infection is hampered by the lack of appropriate animal models for efficacy testing of candidate vaccines. Previously, we have successfully tested the immunogenicity and protectiveness of a candidate EV71 vaccine, containing recombinant Newcastle disease virus capsids that display an EV71 VP1 fragment (NPt-VP1_1-100_) protein, in a mouse model of EV71 infection. A drawback of this system is its limited window of EV71 susceptibility period, 2 weeks after birth, leading to restricted options in the evaluation of optimal dosing regimens. To address this issue, we have assessed the NPt-VP1_1-100_ candidate vaccine in a hamster system, which offers a 4-week susceptibility period to EV71 infection. Results obtained showed that the NPt-VP1_1-100_ candidate vaccine stimulated excellent humoral immune response in the hamsters. Despite the high level of antibody production, they failed to neutralize EV71 viruses or protect vaccinated hamsters in viral challenge studies. Nevertheless, these findings have contributed towards a better understanding of the NPt-VP1_1-100_ recombinant protein as a candidate vaccine in an alternative animal model system.

## Findings

The virion protein 1 (VP1) of EV71 was widely used in vaccine development using different delivery systems and shown to confer protection against lethal EV71 infection in mice [1,2]. The N-terminal portion of the VP1 protein was suggested to contain a major antigenic site [3]. It also preferentially bound by high-titered neutralizing antibodies to EV71 in human cord sera [4]. Antigenic and neutralization determinants, which are important for vaccine development, are thus likely to be located in this region. Sivasamugham and colleagues (2006) developed and studied the first 100 amino acid residues of this N-terminal region of VP1 by fusing it to a carrier protein, a truncated nucleoprotein (NP) of Newcastle disease virus (NDV). This protein construct was designated as NPt-VP1_1-100_ and found to be highly immunogenic in rabbit. The construct was also able to self-assemble into ring-like particles that could increase the VP1_1-100_ immunogenicity. Rabbit sera generated after immunization was shown to recognize and react with the authentic EV71 [3]. Altogether, these data suggest that the recombinant protein has great potential as a promising vaccine candidate against EV71 infections.

Recently, a mouse model with a prolonged susceptibility period to EV71 infections has been developed by Ong and colleagues [5]. The susceptibility period was found to be up to 2-weeks after birth. This well-characterized mouse model infected by a mouse-adapted EV71 strain P5 (EV71^P5^) shared many characteristics with the human central nervous system (CNS) disease. To test the protective efficacy of the NPt-VP1_1-100_ in this mouse model, we initially tested its immunogenicity in mice. It was found to be a potent immunogen in adult mice [6]. Based on these information, we performed an extensive viral challenge studies in the above newborn mouse model. Newborn mice vaccinated with the NPt-VP1_1-100_ showed more than 40 % increase in survival rate compared to the control group [7]. Interestingly, 50 % of these mice fully recovered from their paralysis. Further analyses to improve the protective efficacy of the NPt-VP1_1-100_ were hampered by the fact that the newborn mouse model can only provide a 2-week susceptibility period to EV71 infections [5]. This narrow time window led to limitations in the testing of various parameters for vaccination and viral challenge. Previous study suggested that intervals between doses, age at priming and at the last dose of vaccination are factors that influence infant antibody responses [8]. To address this issue, we needed an animal model which can offer a prolonged susceptibility period. It was observed that in newborn hamsters, EV71 virus was able to cause symptomatic infection for up to 4 weeks after birth (Prof. K.T. Wong, personal communication). Previously, Syrian hamsters were used to study Bulgarian strains of EV71 [9]. The virus caused poliomyelitis-like lesions in their central nervous system, myositis and paralysis. In the present study, we evaluated the immunogenicity and protective efficacy of the EV71 NPt-VP1_1-100_ candidate vaccine in a Syrian hamster model which offers a prolonged period of susceptibility to EV71 infection.

Initially, the full length NP (NPfl) and NPt-VP1_1-100_ recombinant proteins were induced and purified as described previously [6,7]. The EV71^P5^ was obtained from the Department of Pathology, Faculty of Medicine, Universiti Malaya, Malaysia [5]. Purified VP1 protein of Enterovirus 71 was kindly provided by Prof. M.J. Cardosa of the Institute of Health and Community Medicine, Universiti Malaysia Sarawak [4].

Pregnant Syrian hamsters were purchased from the Animal House, Universiti Kebangsaan Malaysia. Animal experiments were done according to The Universiti Putra Malaysia Animal Care and Use Committee guidelines (AUP No: 10R84) and animals were cared for in accordance with The Code to Care and Use of Animals in Research. Newborn hamsters were immunized with three doses (10 μg per dose) of either NPfl (control group; n = 4) or NPt-VP1_1-100_ (n = 5) proteins at 1-, 6- and 13-day old. The first dose containing 50 % Freund’s adjuvant (Sigma, USA) was injected subcutaneously, and subsequent doses containing Freund’s incomplete adjuvant (Sigma, USA) were injected intraperitoneally. On day 28 after birth, all the hamsters were challenged intraperitoneally with 2.53 x 10^6^ TCID_50_ of EV71^P5^ virus. This viral dose was 4 times higher than the dose used in the mouse model study [7]. Their body weight and paralysis score were monitored and recorded daily until day 12 post-challenge. The paralysis score was defined as: score 0, no hind limb paralysis and healthy; score 1, mild paralysis, weakness in hind limb(s); score 2, moderate paralysis, jerky movement; score 3, a hind limb shows severe paralysis; score 4, both hind limbs show severe paralysis. Their survival rate was monitored until the end of the experiment on day 18 post-challenge when they were sacrificed via cardiac puncture to collect their sera for further analysis. All the collected sera were subjected to an indirect enzyme-linked immunosorbent assay (ELISA) and immunoblotting analysis against purified full length VP1 and NPfl proteins as described previously [6,7].

To test for potential EV71 neutralization properties of the antibodies produced, the sera were mixed with an equal volume of 100 TCID_50_ EV71 strain A104 virus and incubated at 37°C for 2 h. The resulting mixtures were assayed on Vero cells seeded in a 96-well plate as described in Ch'ng et al. [7]. Cytopathic effects (CPE) were examined after 7 days of incubation and neutralization titers were determined as the highest dilutions that resulted in a 50 % inhibition of CPE. All the experimental data in this study were analysed using the Student's *t*-test and presented as mean ± standard error (SE). Differences with p <0.05 were considered significant.

**Figure 1:**
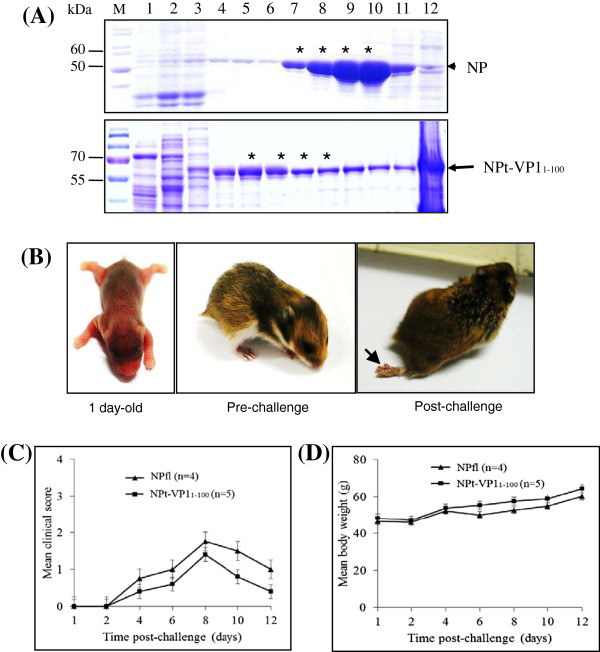
**Protein production and viral protection study.** (**A**) Sedimentation profiles of purified NPfl and NPt-VP1_1-100_ proteins were analyzed on a 12 % SDS-PAGE gel stained with Coomassie Brilliant Blue. * indicates the fractions that were pooled and concentrated. (**B**) Hamster's physiology before and after viral challenge. Arrow indicates limb paralysis. (**C**) Paralysis score and (**D**) body weight of hamsters following challenge with EV71^P5^.

Following purification, the NPfl and NPt-VP1_1-100_ proteins appeared as distinct bands on Coomassie Brilliant Blue-stained 12 % SDS-PAGE gel. As expected, the NPfl and NPt-VP1_1-100_ proteins showed an approximate molecular weight of 55 and 60 kDa, respectively (Figure 1A). Band patterns obtained were similar to our previous reports [3,6,7]. The purified proteins were then used to vaccinate newborn hamsters as described above. Upon challenge with EV71^P5^, the hamsters in both groups showed mild or moderate paralysis symptoms (Figure 1B). The NPt-VP1_1-100_-immunized group, however, showed lower paralysis score compared to the control group (Figure 1C). This lower score correlated with their higher overall weight gain (Figure 1D). These results suggest that even though EV71^P5^ is a mouse-adapted strain [5], it is still capable of infecting hamsters and produce symptoms similar to the ones observed in mice [7], although at a lower severity. In the mice study, we managed to give only 2 doses of vaccination prior to virus challenge due to the 2-week susceptibility period of the mice to EV71 infection. In the present study, we gave 3 doses of vaccination to the hamsters. Despite the increase in dosages and immunization period, the NPt-VP1_1-100_-immunized hamsters failed to display any protection from the EV71^P5^ challenge. Even though they displayed symptoms of EV71 infection, all hamsters including the NPfl control group, survived until the end of experiment. Their body weight increased gradually and their symptoms did not change significantly until the end of the experiment. This is in contrast to the findings obtained from the mouse model study [7]. In that study, a 40% survival rate was observed in the NPt-VP1_1-100_-immunized mice while 100% mortality was seen in the NPfl control mice.

The differences observed between the two animal systems were perhaps due to the use of the EV71^P5^ in the challenge studies. The mouse-adapted EV71^P5^ virus may not be suitable for the hamster model. It was noted that hamsters may only get severe infections and increased mortality when higher doses of virus, compared to the one in the present study, were used (unpublished observation). It is known that virus adaptation to a specific host will alter their infectivity to other hosts. Adaptation of EV71 clinical isolate in Chinese hamster ovary cells resulted in a reduced virulence in newborn BALB/c mice [10]. Differences in genetic background of hosts are also important in viral infections [11]. Receptor specificity is another factor which determines virus cell tropism [12]. Hence, in the present study, the use of the mouse-adapted EV71^P5^ may contribute to the reduced susceptibility in the hamsters.

**Figure 2:**
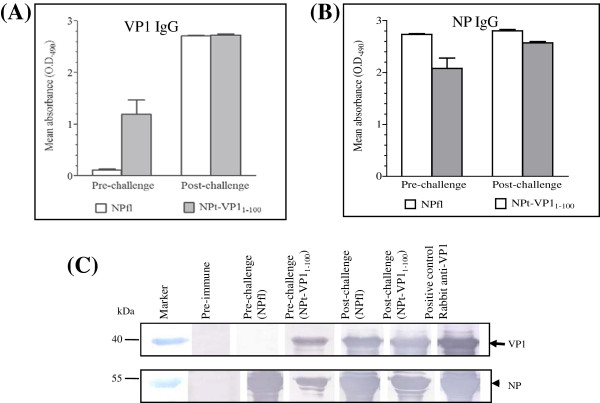
**Anti-VP1 and anti-NP IgG levels in sera before and after viral challenge.** Determination of total anti-VP1 IgG antibodies (**A**) and total anti-NP IgG antibodies (**B**). (**C**) Purified VP1 or NP proteins were separated on 12% SDS-PAGE gel and electro-transferred onto membranes. Strips of the membranes were incubated with different types of sera. Arrow indicates the expected position for VP1 band. Arrowhead indicates the expected position for NPfl band.

Pre-challenge and post-challenge sera from all the hamsters were collected and analyzed using ELISA. When the full length VP1 was used as the coating antigen, no IgG response was observed in the control NPfl hamsters (Figure 2A). In contrast, a remarkable increase (p < 0.0001) was seen in the hamster group vaccinated with the NPt-VP1_1-100_. After viral challenge, both of the groups showed high titers of anti-VP1 IgG. Similar to the results of the mouse studies [6,7], the NPt-VP1_1-100_ was able to induce strong immune responses *in vivo*. In both the NPt-VP1_1-100_ and the NPfl control groups, all hamsters produced extremely high titer of anti-NP before and after viral challenge (Figure 2B). This observation is in agreement with previous studies which showed the highly immunogenic nature of the NP [3,7].

To further confirm the presence of anti-VP1 and anti-NP antibodies in all the collected sera, immunoblotting was performed. The sera were assayed against separated, purified full length VP1 and NPfl proteins. An intense band of about 40 kDa appeared on the VP1 membrane indicating positive detection for anti-VP1 antibodies (Figure 2C, arrow). As observed in the ELISA study, no band was noted in the pre-immune, as well as the pre-challenge sera from the control group. On the NPfl membrane, a band with approximate size of 55 kDa formed, confirming the presence of anti-NP antibodies (Figure 2C, arrowhead). The absence of a band in the pre-immune sera corroborated the previous ELISA findings. These results showed that the NPt-VP1_1-100_ was capable of inducing high levels of immune responses in hamster. Overall, the immunogenicity of the protein is high in rabbit [3], mice [7] as well as hamsters.

**Figure 3:**
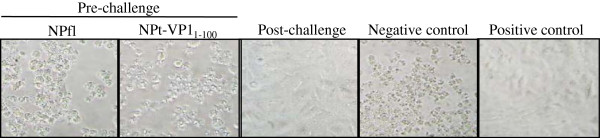
**Cytopathic effects in Vero cells following a neutralization test.** Mixtures of sera dilutions and EV71 strain A104 virus were assayed on Vero cells. Cytopathic effects were examined after 7 days of incubation and neutralization titers were determined. Pre-challenge sera showed the NPfl-immunized samples (1:8 dilution) and NPt-VP1_1-100_-immunized samples (1:8 dilution). 1:512 dilution was used for the post-challenge and the positive control sera. No serum was used in the negative control samples. Magnification = 200X.

To investigate whether the antibodies produced were able to neutralize EV71 virus, neutralization assay was performed. Following seven days of incubation, CPE in Vero cells was observed in the pre-challenged NPfl and NPt-VP1_1-100_ samples. Representative images of the CPE are shown in Figure 3. In all the post-challenge sera samples, no CPE was observed up to 1:512 titers. This finding is in line with the results found in the mice studies [7]. The antibodies against NPt-VP1_1-100_ produced in mice also failed to neutralize EV71 virus despite partially protecting the mice against a lethal viral challenge.

Results obtained in this study showed that the NPt-VP1_1-100_ candidate EV71 vaccine was capable of providing excellent immune stimulation in newborn hamsters. These data provide additional evidence that NPt-VP1_1-100_ is a promising EV71 vaccine candidate. In addition, our findings also suggest that the hamster system may be used as an alternative animal model for the efficacy testing of EV71 candidate vaccines. Since the lack of appropriate animal models is one of the major hurdles for development of an effective vaccine towards EV71 infection, our findings will contribute towards the information needed for finding an optimum animal model. Importantly, the hamster system offers a prolonged susceptibility period to EV71 infection which allows for a more flexibility in parameter testing for vaccination and viral challenge. To improve this hamster system, we are currently optimizing several parameters such as the amount of proteins used in the vaccination, the immunization doses and intervals and the virus strain used in the challenge experiment. Nevertheless, these findings have paved ways towards a more comprehensive study of the evaluation of NPt-VP1_1-100_ recombinant protein as a candidate EV71 vaccine in an alternative animal model system.

## Abbreviations

EV71, Enterovirus 71; VP1, Virion protein 1; NP, Nucleoprotein; NPt-VP1_1-100_, N-terminal region of VP1 containing 100 amino acid residues fused with a truncated NP of NDV; NPfl, Full length NP; CNS, Central nervous system; EV71^P5^, Mouse-adapted EV71 strain P5; CPE, Cytopathic effects.

## Competing interests

The authors declare that they have no competing interests.

## Authors’ contributions

NS, EJS, KY, KCO, KTW designed this study and revised the manuscript critically; WCC carried out this study and drafted the manuscript. All of the authors read and approved the final version of this manuscript.
